# Ranolazine Attenuates Trastuzumab-Induced Heart Dysfunction by Modulating ROS Production

**DOI:** 10.3389/fphys.2018.00038

**Published:** 2018-02-06

**Authors:** Gennaro Riccio, Salvatore Antonucci, Carmela Coppola, Chiara D'Avino, Giovanna Piscopo, Danilo Fiore, Carlo Maurea, Michele Russo, Domenica Rea, Claudio Arra, Gerolama Condorelli, Fabio Di Lisa, Carlo G. Tocchetti, Claudia De Lorenzo, Nicola Maurea

**Affiliations:** ^1^Department of Pharmacy, Federico II University, Naples, Italy; ^2^Department of Biomedical Sciences and CNR Institute of Neuroscience, University of Padova, Padova, Italy; ^3^Division of Cardiology, National Cancer Institute, G. Pascale Foundation, Naples, Italy; ^4^Department of Molecular Medicine and Medical Biotechnology, Federico II University, Naples, Italy; ^5^CEINGE Biotecnologie Avanzate, Naples, Italy; ^6^Department of Translational Medical Sciences, Federico II University, Naples, Italy; ^7^Department of Animal Experimental Research, National Cancer Institute, G. Pascale Foundation, Naples, Italy

**Keywords:** trastuzumab cardiotoxicity, ranolazine, heart function, heart failure, oxidative stress

## Abstract

The ErbB2 blocker trastuzumab improves survival in oncologic patients, but can cause cardiotoxicity. The late Na+ current inhibitor ranolazine has been shown to counter experimental HF, including doxorubicin cardiotoxicity (a condition characterized by derangements in redox balance), by lowering the levels of reactive oxygen species (ROS). Since ErbB2 can modulate ROS signaling, we tested whether trastuzumab cardiotoxicity could be blunted by ranolazine via redox-mediated mechanisms. Trastuzumab decreased fractional shortening and ejection fraction in mice, but ranolazine prevented heart dysfunction when co-administered with trastuzumab. Trastuzumab cardiotoxicity was accompanied by elevations in natriuretic peptides and matrix metalloproteinase 2 (MMP2) mRNAs, which were not elevated with co-treatment with ranolazine. Trastuzumab also increased cleavage of caspase-3, indicating activation of the proapoptotic machinery. Again, ranolazine prevented this activation. Interestingly, Neonatal Rat Ventricular Myocytes (NRVMs), labeled with MitoTracker Red and treated with trastuzumab, showed only a small increase in ROS compared to baseline conditions. We then stressed trastuzumab-treated cells with the beta-agonist isoproterenol to increase workload, and we observed a significant increase of probe fluorescence, compared with cells treated with isoproterenol alone, reflecting induction of oxidative stress. These effects were blunted by ranolazine, supporting a role for *I*Na inhibition in the regulation of redox balance also in trastuzumab cardiotoxicity.

## Introduction

ErbB2 (also called HER2) is tyrosine kinase receptor, member of the human epidermal growth factor receptor family, and is overexpressed in 25–30% of breast cancers (Slamon et al., 1987). Trastuzumab is the prototypical anti-ErbB2 drug, and the first developed and most widely used biologic anticancer agent. Since its introduction in 1998, trastuzumab has dramatically improved the clinical history of breast cancer patients, but unfortunately it has been shown to cause cardiac dysfunction (Bloom et al., 2016; Moslehi, 2016; Zamorano et al., 2016; Armenian et al., 2017), since ErbB2 has been proven to be an important modulator of myocardial function (Force et al., 2007; Eschenhagen et al., 2011). Indeed, in the heart, ErbB2 seems to mediate cell survival and functionality (De Keulenaer et al., 2010; Ky et al., 2013; Lim et al., 2015), and also cardiac regeneration (D'Uva et al., 2015), and it seems to be stimulated upon cardiac adverse hemodynamics or other stress, such as doxorubicin (De Keulenaer et al., 2010; Tocchetti et al., 2017). It has been hypothesized that ErbB2 blockers can hamper cardiomyocytes, especially when exposed to other stressors, such as pressure or volume overload or anthracyclines, eventually leading to cardiac dysfunction (de Korte et al., 2007; Gabrielson et al., 2007; Ewer and Ewer, 2010). Hence, this co-administration is now possibly avoided in clinics (Slamon et al., 2001; Suter et al., 2007), since trastuzumab can exacerbate or induce anthracycline toxicity: once trastuzumab blocks the protective mechanisms of ErbB2, the oxidative damage from anthracyclines can increase (Ewer and Ewer, 2010). Importantly, ErbB2 has been shown to modulate doxorubicin-induced redox damage (Timolati et al., 2006), while its blockade is able to induce myocytes death through redox-dependent pathways (Gordon et al., 2009). In addition, ErbB2 overexpressor mice showed upregulation of antioxidant enzymes and protection from anthracyclines cardiotoxicity (Belmonte et al., 2015).

The late Na+ current inhibitor ranolazine has emerged as a potential therapeutic to treat experimental heart failure (Sabbah et al., 2002; Rastogi et al., 2008), and has also been recently indicated as a promising cardio-oncological drug (Minotti, 2013). We and others (Tocchetti et al., 2014; Cappetta et al., 2017) have demonstrated that ranolazine is also able to blunt experimental doxorubicin cardiotoxicity. Also, we had previously shown that trastuzumab can cause experimental heart dysfunction (Riccio et al., 2009; Fedele et al., 2012). Hence, here we hypothesize that ranolazine is also able to blunt heart dysfunction induced by trastuzumab in animal and cellular models.

## Materials and methods

### Trastuzumab treatment protocol *in vivo*

C57Bl/6 mice (2–4 months old, Harlan Italy, San Piero al Natisone, Udine, Italy) were injected with a cumulative dose of 2 nM trastuzumab (Genentech, South San Francisco, CA, USA) via seven daily intraperitoneal injections (2.25 mg/kg i.p., TRA group), as for our well-established protocol (Fedele et al., 2012). No mortality was associated with this dosing regimen. Another group of mice was treated orally with ranolazine (Ranexa, Menarini, 305 mg/kg/day, doses comparable Reagan-Shaw et al., 2008 with those used clinically in humans of 750 mg twice daily, below the human maximal dosing of 1 g twice daily) for 10 days (RAN group; Tocchetti et al., 2014), and another group, after 3 days of ranolazine, started receiving trastuzumab concomitantly with ranolazine for 7 days (RAN+TRA group). Sham animals were used as controls. For *ex vivo* analyses, animals were sacrificed by cervical dislocation after anesthesia with tilotamine (0.09 mg/g), zolazepam (0.09 mg/g), and 0.01% atropine (0.0 4 ml/g); hearts were then excised and processed for further studies. Eight to ten animals per group were studied for all protocols.

### Transthoracic echocardiography

*In vivo* cardiac function was assessed by transthoracic echocardiography in sedated 2- to 4-month-old WT C57BL6 mice (Harlan Italy, San Piero al Natisone, UD, Italy) using a Vevo 2100 high-resolution imaging system (40-MHz transducer, VisualSonics, Toronto, ON, Canada). Mice were anesthetized with Tilotamine (0.09 mg/g), Zolazepam (0.09 mg/g), and 0.01% atropine (0.04 ml/g). Cardiac function was evaluated by non-invasive echocardiography in basal conditions, after 7 days of treatment with trastuzumab, or after 3 days of pre-treatment with ranolazine followed by co-administration of ranolazine and trastuzumab for 7 days. Studies and analysis were performed blinded to heart condition. Data are presented as mean ± standard error of the mean (SEM) unless otherwise noted. Between-group differences were assessed by Student's *t*-test or one-way analysis if variance (ANOVA) as appropriate. Statistical significance was defined as *P* < 0.05.

The animal experiments described herein were carried out in accordance with the recommendations of Italian regulations for experimentation on animals. The protocol was approved by the ethical committee and met the standards required by Directive 2010/63/EU of the European Parliament.

### RNA extraction and real-time PCR

Fresh frozen tissue was mechanically homogenized and total RNA was extracted by using Trizol (Invitrogen, Milan, Italy) according to the manufacturer's protocol. Reverse transcription of total RNA was performed starting from equal amounts of total RNA/sample (500 ng) using SuperScript® III Reverse Transcriptase (Invitrogen, Milan, Italy). RT-PCR was used to assess the mRNAs of ANP, MMP2, and GAPDH (the latter as an internal reference), as previously described (Tocchetti et al., 2014). Experiments were carried out in triplicate for each data point, and data analysis was performed with Applied Biosystems'StepOnePlus™ Real-Time PCR System.

### Western blotting analysis of the apoptotic pathway

Murine hearts were processed as previously described (Tocchetti et al., 2014). Anti-caspase 3, anti-cleaved caspase 3, anti-GAPDH (Cell Signaling Technology), or anti-Actin antibody (Sigma), followed by anti-rabbit, HRP-conjugated IgGs from goat antiserum (Thermo Scientific) were used to detect proteins involved in the apoptotic pathway. The signal from secondary antibodies was visualized by enhanced chemiluminescence detection (ECL western blotting detection kit, Thermo Scientific). The signal intensity of reactive bands was quantitatively measured with a phosphorimager (GS-710, Biorad) or by the open source software ImageJ (NIH, USA).

### Isolation and culture of neonatal rat ventricular myocytes (NRVMs)

NRVMs were isolated from 1 to 3 days old Wistar rats. Hearts were excised, fragmented and dissociated at 4°C overnight with an enzyme solution containing trypsin (Invitrogen), under continuous stirring. The day after, fragments were further dissociated with an enzymatic solution containing Collagenase type I (Gibco). Cells were purified and resuspended in growth medium consisting of MEM (Invitrogen) supplemented with 10% fetal bovine serum (Gibco) and antibiotics (100 U/ml penicillin and 100 μg/ml streptomycin).

Cells were pre-plated for 90 min in order to separate NRVMs from fibroblasts. NRVMs were counted and opportunely diluted with MEM supplemented with 10% FBS, antibiotics, non-essential amino acids (NEAA) and 0.1 mM BrdU, necessary to inhibit cell proliferation, and the plated onto slides pre-treated with gelatin 0.1%.

### Measurement of formation of reactive oxygen species

NRVMs were seeded in 6-well plates at a density of 3 × 10^5^/well and ranolazine (10 μM) and trastuzumab (2 μM), alone or in combination, were added.

After 24 h, cells were incubated for 15 min at 37°C with 10 nM MitoTracker Red CM-H2XRos (MTR, Molecular Probes, λec = 579 nm, λem = 599 nm) in HBSS. Following the incubation, cells were washed twice with HBSS and slides were analyzed using the fluorescence microscope Zeiss Axlovert 100 M and a 63x oil immersion objective.

In the kinetic experiment, NRVMs were treated with ranolazine (10 μM) and trastuzumab (2 μM), alone or in combination. After 24 h, cells were incubated with 10 nM MitoTracker Red CM-H_2_XRos as shown before. Three baseline images were taken at frame rate 1/5 min, and then 4 μM Isoproterenol (ISO) was added for 1 h.

### Statistical analyses

For most studies, between-group differences were assessed by Student's *t*-test or one-way ANOVA as appropriate. Statistical analysis was performed with OriginPro 8 SR0 v8.0724. Differences among the groups in parameters assessed by reverse transcription–PCR (RT–PCR) or western blotting were evaluated using the non-parametric Kruskal–Wallis test and adjusted for multiple comparisons with the Bonferroni method Statistical analysis were performed with SPSS statistical package (14.0 version).

Statistical significance was defined as *P* < 0.05.

## Results

### Ranolazine attenuates trastuzumab-induced heart dysfunction in mice

First, we tested the beneficial role of ranolazine on trastuzumab induced cardiotoxicity *in vivo* in a mouse model. To this aim, groups of 8–10 mice were treated with trastuzumab and ranolazine as described in the Methods section. Echocardiography was performed before and after the treatments.

**Figure 1:**
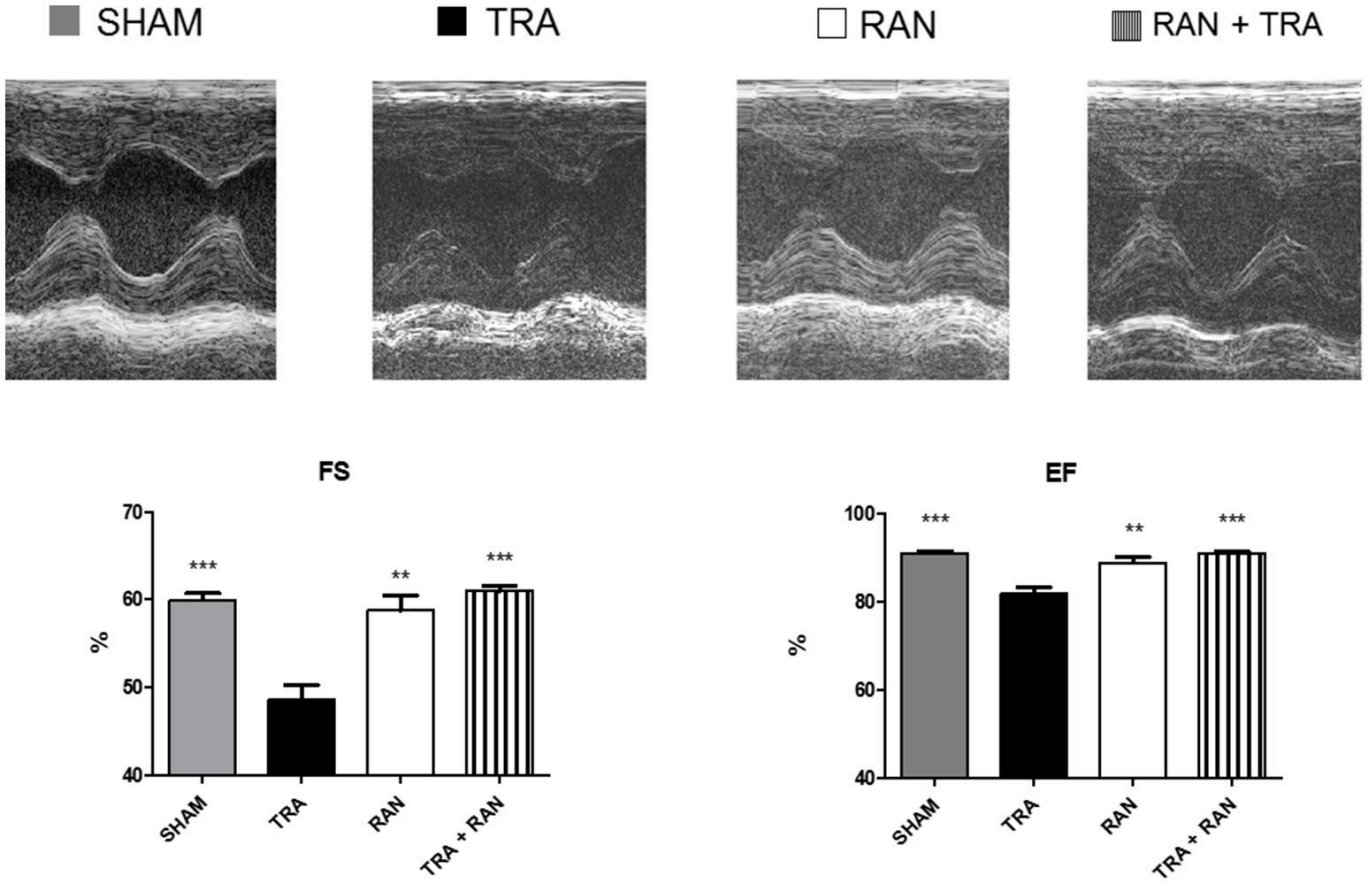
Ranolazine protects from trastuzumab-related cardiac dysfunction. Ranolazine blunts reductions in fractional shortening (FS) and ejection fraction (EF) induced by trastuzumab (^**^p < 0.01 vs TRA; ^***^p < 0.001 vs TRA).

After 7 days, in trastuzumab-treated mice, fractional shortening (FS) decreased to 54 ± 3.86%, *p* < 0.0005 vs. 60.29 ± 2.47% (sham), ejection fraction (EF) to 84.33 ±3.08%, *p* < 0.0005 vs. 91.26 ± 2.03% (sham). However, in mice treated with ranolazine plus trastuzumab, FS and EF were not significantly reduced (FS 59.40 ± 3.13%; EF 89.80 ± 2.28%; Figure 1).

### Ranolazine prevents cardiac fetal gene reprograming and extracellular matrix remodeling in trastuzumab treated hearts

**Figure 2:**
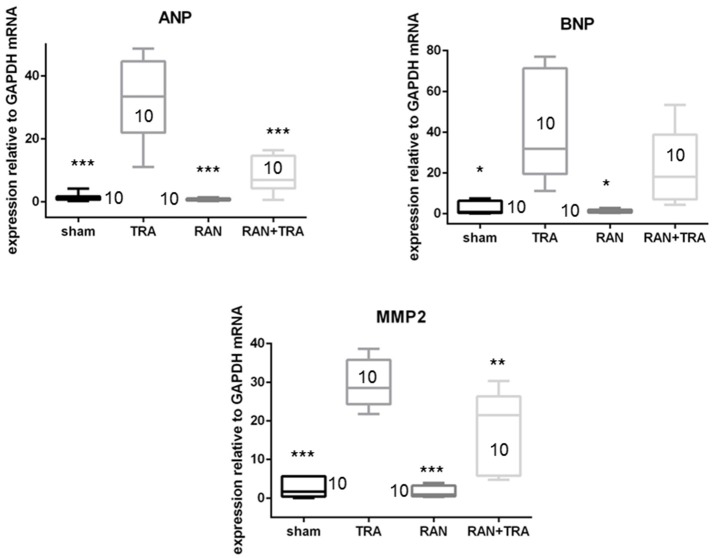
Effects of ranolazine on cardiac fetal gene reprogramming. ANP, BNP, and MMP2 mRNA expressions induced by trastuzumab were reduced in mice pretreated with ranolazine (^*^p < 0.05 vs. TRA; ^**^p < 0.005; ^***^p < 0.0005).

At the end of the *in vivo* treatment, mice were euthanized, and hearts were removed and processed for mRNA expression analyses and detection of myocardial stress and apoptosis. All analyses were performed in parallel experiments on sham animals. In accordance with the alterations of contractile function, trastuzumab enhanced ANP and MMP2 mRNAs compared with sham. However, when mice were pre-treated with ranolazine, ANP, and MMP2 mRNA levels were lower compared to TRA (Figure 2).

### Ranolazine reduces trastuzumab-induced apoptosis

**Figure 3:**
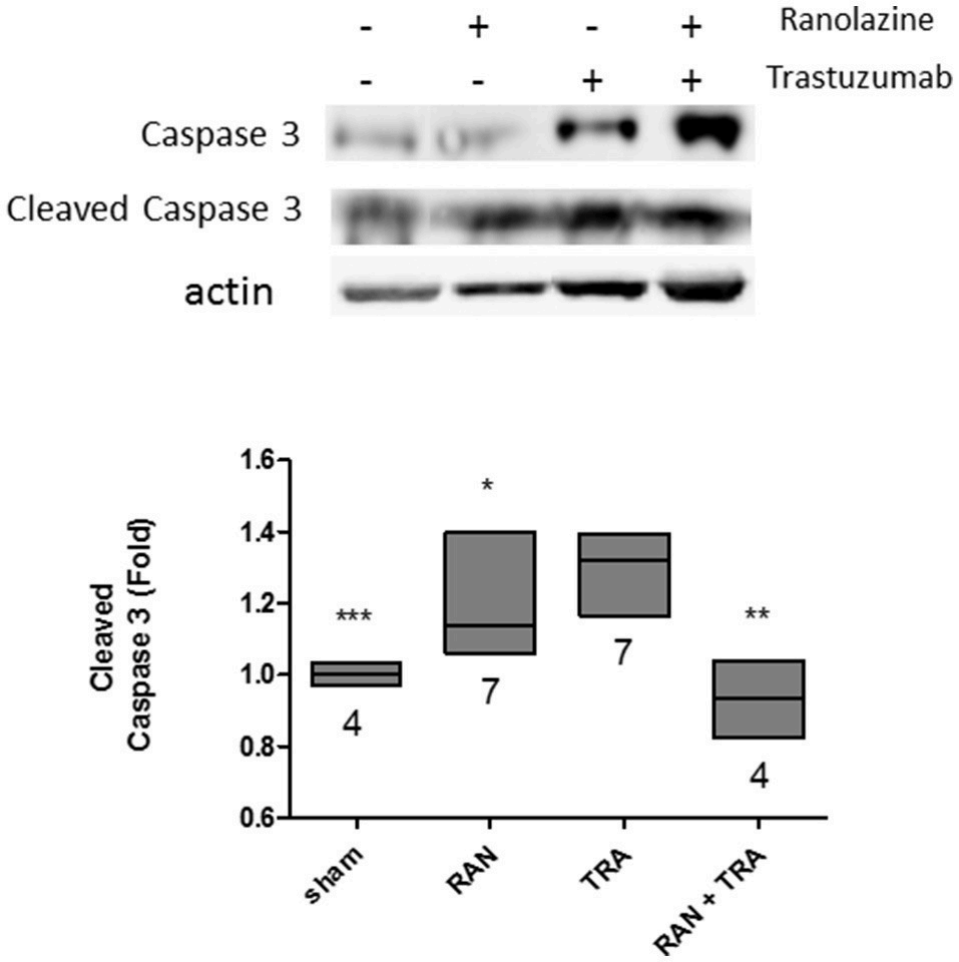
Apoptotic pathway analysis in hearts of treated mice. Caspase 3 activation triggered by trastuzumab, was reduced by ranolazine treatment (^*^p < 0.05 vs. TRA; ^**^p < 0.005 vs. TRA; ^***^p < 0.0005 vs. TRA).

We then investigated the impact of ranolazine on trastuzumab-induced cell death. Excised murine hearts of treated mice were handled as described above, then lysed and analyzed by Western blotting with an anti-Caspase antibody. Trastuzumab activated apoptosis significantly, as shown by the enhanced cleavage of caspase-3. Caspase-3 fragmentation did not occur when mice were co-administered with RAN+TRA (Figure 3).

### Ranolazine limits the production of reactive oxygen species induced by trastuzumab in NRVMs

To test whether ranolazine protective effects are achieved by a reduction of ROS generated upon trastuzumab administration, ROS formation was monitored in NRVMs. NRVMs labeled with MitoTracker Red and treated for 24 h with Trastuzumab showed a modest increase of probe fluorescence, compared with untreated cells, reflecting induction of oxidative stress, which was not blunted by ranolazine.

**Figure 4:**
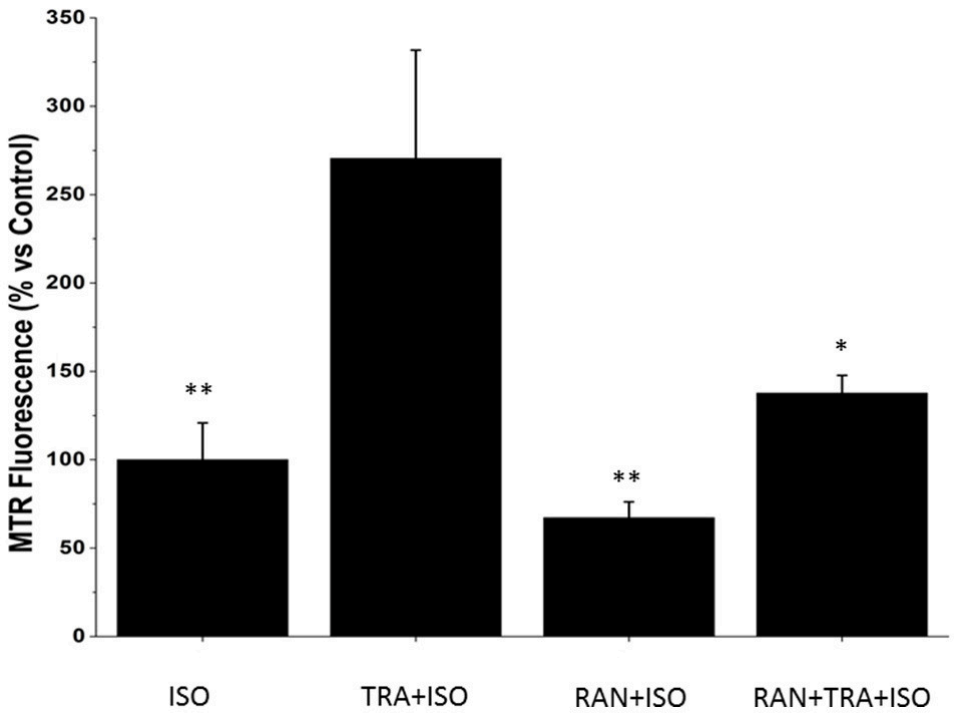
Ranolazine (RAN) reduces Reactive Oxygen Species (ROS) formation induced by Trastuzumab (TRA) and isoproterenol (ISO). Neonatal Rat Ventricular Myocytes (NRVMs) untreated or treated for 24 h with 2 μM TRA, with 10 μM Ran or with 2 μM TRA and 10 μM Ran, were incubated for 1 h with 4 μM Iso. Microscopy analysis of cells labeled with the fluorescent probe Mitotracker Red CM-H2XRos was performed to detect the degree of mitochondrial oxidative stress. Data are normalized to the control after 24 h + 1 h of treatment with isoproterenol. n ISO: 24; n TRA+ISO: 22; n RAN+ISO: 19; n RAN+TRA+ISO: 20. ^*^p < 0.05 vs. TRA; ^**^p < 0.01 vs. TRA.

Considering that trastuzumab exerts its cardiotoxic effects especially in presence of cardiac stressors (de Korte et al., 2007; De Keulenaer et al., 2010; Ewer and Ewer, 2010; Tocchetti et al., 2017), and that the beating heart is normally subject to preload and afterload, we then incubated NRVM with the beta stimulator isoproterenol (ISO; Tocchetti et al., 2012) to simulate workload conditions. Interestingly, the concomitant administration of TRA and ISO produced a significant increase in ROS compared to ISO alone, and such increase could be blunted by RAN (Figure 4).

## Discussion

The introduction of trastuzumab in therapeutic protocols for ErbB2+ breast cancer has revolutionized the prognosis of patients affected by this disease, but unfortunately this therapy is characterized with a relevant incidence of cardiac dysfunction and HF, especially when associated with anthracyclines. We (Tocchetti et al., 2014) and others (Cappetta et al., 2017) have shown that the late *I*Na inhibitor ranolazine is able to blunt cardiac dysfunction induced by anthracyclines by decreasing ROS production. The data presented here suggest that ranolazine is also able to blunt trastuzumab cardiotoxicity, and this effect seems to involve a reduction in oxidative stress. Indeed, redox mechanisms have also been proposed for the neuregulin/ErbB2 pathway. This pathway has been shown to play a role in modulating the increase in ROS caused by doxorubicin in animal models (Timolati et al., 2006), suggesting that cardiotoxicity from ErbB2 blockade can also involve a dysregulation of redox homeostasis (Mercurio et al., 2016). Importantly, beside anthracyclines (Menna et al., 2012; Sawyer, 2013; Stěrba et al., 2013; Ghigo et al., 2016), redox abnormalities are involved in the pathophysiology of cardiotoxic effects caused by several antineoplastic drugs (Ferroni et al., 2011), including new biologic anti-cancer drugs, such as intracellular signaling inhibitors, that are increasingly used in recent years (Tocchetti et al., 2017). Such drugs may be cardiotoxic, since they block pathways that are major modulators of myocardial function, especially under conditions of cardiac stress, such as hypertension or hypertrophy (Suter and Ewer, 2013), with mechanisms of action that often involve redox dysregulation as well.

The importance of ErbB2 in the heart has been particularly emphasized by seminal studies that demonstrated that ErbB2 cardiac KO mice were affected by dilated cardiomyopathy, with increased susceptibility to anthracycline-induced damage to cardiomyocytes (Crone et al., 2002; Ozcelik et al., 2002). Conversely, cardiac ErbB2 overexpressor mice showed lower levels of ROS in mitochondria, with reduced ROS levels and less cell death in neonatal cardiomyocytes isolated from ErbB2(tg) hearts upon administration of anthracyclines, due to increased levels of glutathione peroxidase 1 (GPx1) protein and GPx activity, with enhanced levels of two known GPx activators, c-Abl, and Arg (Belmonte et al., 2015; Tocchetti et al., 2017). Furthermore, block of ErbB2 has been correlated with cardiomyocyte death through reactive oxygen species-dependent pathways (Gordon et al., 2009).

Along this line, our results in NRVM show that attenuation of trastuzumab toxicity with ranolazine is indeed obtained by reducing ROS production, and our *in vivo* data show better LV function with ranolazine+trastuzumab compared with trastuzumab alone. The fact that trastuzumab elicited only a modest rise in ROS in non-stressed NRVM is compatible with the cardiotoxic effect of ErbB2 blockers that might be negligible *per se*, but exacerbated when administered under conditions of cardiac stress or in previously diseased hearts (e.g., increased pressure or volume overload) or in presence of cardiovascular risk factors (age, obesity, smoking, hypertension, previous exposure to anthracyclines; Denegri et al., 2016; Tocchetti et al., 2017).

The inhibition of late INa with ranolazine has been proposed as a therapeutic strategy in many *in vivo* and *in vitro* models of heart dysfunction (Sabbah et al., 2002; Rastogi et al., 2008; Coppini et al., 2013, 2017) and in particular, ranolazine has been shown to be able to blunt LV dysfunction in experimental models of doxorubicin cardiotoxcicity by lowering oxidative stress (Tocchetti et al., 2014; Cappetta et al., 2017). By reducing elevated [Na+]i levels that, are commonly elevated in conditions of cardiac dysfunction (Bers, 2001; Pieske and Houser, 2003), ranolazine could prevent calcium overload and the occurrence of oxidative damage by reducing ROS production, with an advantage over ordinary antioxidant treatments that counteract ROS after their generation (Zeitz et al., 2002; Maack et al., 2006; Song et al., 2006; Wagner et al., 2006; Erickson et al., 2008; Kohlhaas et al., 2010; Tocchetti et al., 2014).

In the setting of heart disease, ROS play a role in pathophysiological remodeling, cellular death, and LV dysfunction (Sawyer et al., 2002; Giordano, 2005; Takimoto and Kass, 2007; Nediani et al., 2011). The molecular signaling pathways that link ROS to LV hypertrophy, remodeling, and failure include alpha- and beta-adrenergic and angiotensin II (AT1) receptor stimulation, as well as modifications of a wide number of proteins that include stress kinases, nuclear transcription factors, collagen and metalloproteinases, calcium channels, myofilaments and proteins involved in the excitation–contraction coupling machinery. A key role is played by a rise in cytosolic Ca2+ levels that lead to expression changes of several genes involved in cardiac pathophysiological hypertrophy and remodeling of the heart (Arcaro et al., 2016) with increases in interstitial fibrosis and expression of profibrotic genes (Zhao et al., 2010). Abnormalities of the extracellular matrix and adverse remodeling are also exacerbated by ROS (Kandasamy et al., 2010). Importantly, our data show that ranolazine is able to blunt the effects produced by trastuzumab on important components of LV remodeling such as myocyte death and fibrosis, and to reverse the expression changes of important genes such as NPs and MMPs, eventually mitigating the occurrence of cardiac dysfunction measured by echocardiography.

## Limitations of the study

In human pathology trastuzumab is administered to cancer patients, while here we studied experimental trastuzumab cardiotoxicity in C57BL6 mice without cancer. Of course, further studies in mice with cancer will have to be performed. Nevertheless, C57BL6 mice, that are commonly used in models of experimental heart failure, also have a compromised immune system that in part may mimic cancer.

## Conclusions

Our data support previous findings on the efficacy of ranolazine in experimental heart dysfunction (Sabbah et al., 2002; Rastogi et al., 2008; Coppini et al., 2013, 2017; Tocchetti et al., 2014; Cappetta et al., 2017). We acknowledge that further experiments may be necessary to conclude that the mechanism of action involves the levels of ROS, also considering that ranolazine has been recently shown to be able to antagonize β-adrenergic stimulation and decrease myofilaments Ca2+ sensitivity (Flenner et al., 2016), with little therapeutic efficacy in a HCM murine model *in vivo*. Nevertheless, we show that in the cardio-oncologic setting, beside doxorubicin cardiotoxicity (Tocchetti et al., 2014; Cappetta et al., 2017), RAN could also be a promising cardioprotective drug in the setting of trastuzumab toxicity. More efforts involving both experimental and clinical studies will be needed in order to establish whether ranolazine might be introduced clinically in the therapeutic strategies that aim at addressing cardiotoxicity induced by trastuzumab or anthracyclines.

## Author contributions

GR, SA, CDA, DF, and CM: performed *in vitro* experiments; CC and DR: performed *in vivo* experiments; GR, SA, CC, GP, DF, MR, DR, and CGT: analyzed data, drafted figures, and the manuscript; CA, GC, FDL, CDL, and NM: provided necessary materials; FDL, CGT, CDL, and NM: conceptualized the project; CGT and CDL: wrote the manuscript.

### Conflict of interest statement

The authors declare that the research was conducted in the absence of any commercial or financial relationships that could be construed as a potential conflict of interest. The handling Editor declared a past co-authorship with one of the authors CGT.
